# Endo180 (*MRC2*) Antibody–Drug Conjugate for the Treatment of Sarcoma

**DOI:** 10.1158/1535-7163.MCT-22-0312

**Published:** 2022-11-18

**Authors:** Rachel J. Evans, Douglas W. Perkins, Joanna Selfe, Anna Kelsey, Gavin P. Birch, Janet M. Shipley, Koen Schipper, Clare M. Isacke

**Affiliations:** 1 The Breast Cancer Now Toby Robins Research Centre, The Institute of Cancer Research, London, UK.; 2 Sarcoma Molecular Pathology Team, Divisions of Molecular Pathology and Cancer Therapeutics, The Institute of Cancer Research, London, UK.; 3 Department of Paediatric Pathology, University of Manchester Foundation Trust, Manchester, UK.; 4 Abzena (Cambridge) Ltd., Babraham Research Campus, Babraham, Cambridge, UK.

## Abstract

Although the 5-year survival rates for sarcoma patients have improved, the proportion of patients relapsing after first-line treatment remains high, and the survival of patients with metastatic disease is dismal. Moreover, the extensive molecular heterogeneity of the multiple different sarcoma subtypes poses a substantial challenge to developing more personalized treatment strategies. From the IHC staining of a large set of 625 human soft-tissue sarcomas, we demonstrate strong tumor cell staining of the Endo180 (*MRC2*) receptor in a high proportion of samples, findings echoed in gene-expression data sets showing a significantly increased expression in both soft-tissue and bone sarcomas compared with normal tissue. Endo180 is a constitutively recycling transmembrane receptor and therefore an ideal target for an antibody–drug conjugate (ADC). An anti-Endo180 monoclonal antibody conjugated to the antimitotic agent, MMAE via a cleavable linker, is rapidly internalized into target cells and trafficked to the lysosome for degradation, causing cell death specifically in Endo180-expressing sarcoma cell lines. In a sarcoma tumor xenograft model, the Endo180-vc-MMAE ADC, but not an isotype-vc-MMAE control or the unconjugated Endo180 antibody, drives on-target cytotoxicity resulting in tumor regression and a significant impairment of metastatic colonization of the lungs, liver and lymph nodes. These data, together with the lack of a phenotype in mice with an *Mrc2* genetic deletion, provide preclinical proof-of-principle evidence for the future development of an Endo180-ADC as a therapeutic strategy in a broad range of sarcoma subtypes and, importantly, with potential impact both on the primary tumor and in metastatic disease.

## Introduction

Sarcomas represent over 100 different cancer subtypes that derive from mesenchymal cells in bone, cartilage, or connective tissues and are broadly categorized as either soft-tissue sarcomas (STS) or bone sarcomas (1, 2). Although sarcomas are relatively rare, causing less than 1% of adult cancers, some subtypes, such as rhabdomyosarcomas, predominantly affect those under 20 year olds. Together, sarcomas are responsible for 12% of childhood and young adult cancers (3). The current standard of care for adults with localized disease is surgery with or without radiation; however, patients with unresectable or metastatic disease also receive chemotherapy and radiotherapy (4). Despite intensive treatment, greater than 50% of patients with high-grade STS develop metastatic disease resulting in a median overall survival of 19 to 20 months (5). The 5-year overall survival for patients with metastatic bone sarcoma is 30% (6, 7). In recent years, there has been renewed activity in using immunotherapies to target sarcoma. In total, 9 immunoconjugates targeting sarcomas have entered clinical trials, 7 antibody–drug conjugates (ADC) and 2 radioimmunoconjugates, although none have yet been approved for clinical use (8). Therefore, despite these advances, there remains a significant unmet clinical need to identify effective therapeutic targets in sarcoma, especially in the metastatic setting.

**Figure 1.**
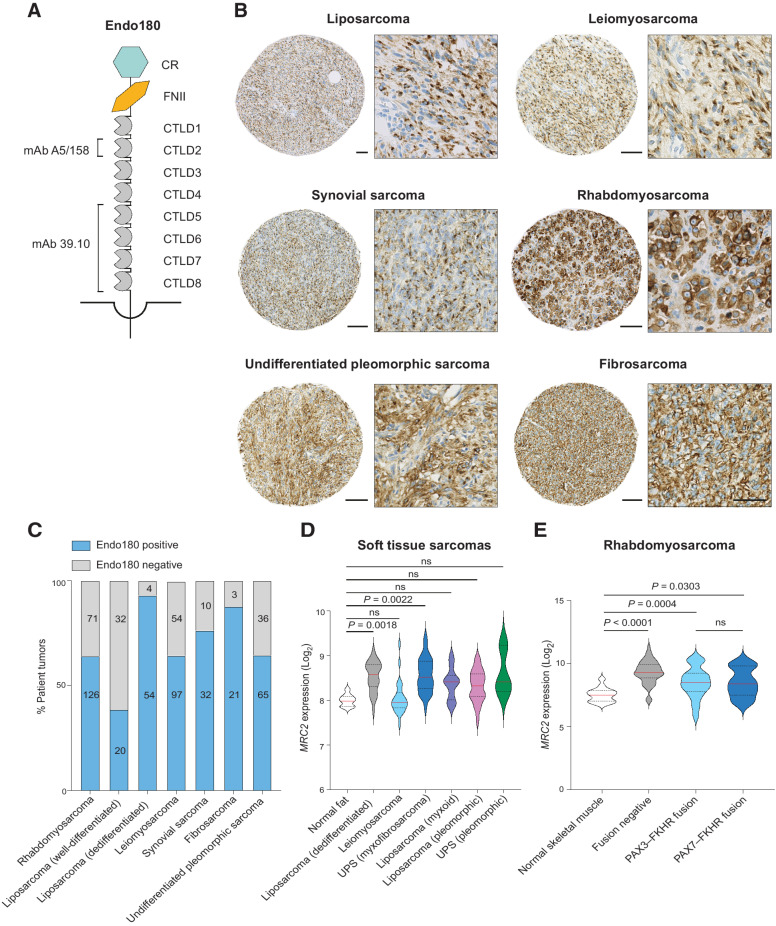
Endo180 is expressed on multiple sarcoma subtypes. **A,** Schematic of the Endo180 protein structure and mAb binding sites. CR, cysteine-rich domain; FNII, fibronectin type II domain; CTLD, C-type lectin-like domains. **B** and **C,** STS tumor microarrays stained with the anti-Endo180 mAb 39.10. **B**, Representative images of Endo180-positive tumor cores from a total of 625 sarcoma patient samples, additional examples shown in Supplementary Fig. S1. Scale bars, 100 μm (whole core), 50 μm (enlarged inset). **C**, Bar chart showing the proportion of tumors scored as Endo180-positive. **D,***MRC2* expression from Barretina et al. (30) in normal fat (*n* = 9), liposarcoma (dedifferentiated; *n* = 46), leiomyosarcoma (*n* = 26), UPS (myxofibrosarcoma; *n* = 31), liposarcoma (myxoid; *n* = 20), liposarcoma (pleomorphic; *n* = 23), and UPS (pleomorphic; *n* = 3; violin plots, red lines indicate median expression levels, dotted lines indicate upper and lower quartiles, Kruskal–Wallis test, Dunn multiple comparison test). **E,***MRC2* expression from ITCC samples in normal skeletal muscle (*n* = 30) and rhabdomyosarcoma grouped as fusion negative (*n* = 56), paired box 3-forkhead (PAX3-FKHR) fusion (*n* = 34), and PAX7–FKHR fusion (*n* = 10; violin plots, red lines indicate median expression levels, dotted lines indicate upper and lower quartiles, one-way ANOVA test, Tukey multiple comparison test).

Endo180 (gene name *MRC2*, also known as uPARAP, CD280, and TEM1) is a member of the mannose receptor family containing an N-terminal cysteine-rich domain, a collagen-binding fibronectin type II domain (9, 10), 8 C-type lectin-like domains (CTLD), a single transmembrane domain and a short cytoplasmic domain that mediates receptor internalization (Fig. 1A; ref. 11). In adults, the expression of Endo180 is largely restricted to normal tissue fibroblasts but the expression is upregulated on cancer-associated fibroblasts (CAF) and particularly the subset of myofibroblastic CAFs (12, 13). Importantly, mice with a whole-body genetic deletion of Endo180 have no overt phenotype (14, 15); however, when implanted with syngeneic tumor cells, they show impaired tumor progression and a reduction in viable CAFs (13). In addition to CAFs, Endo180 receptor expression has also been reported on tumor cells of mesenchymal origin including glioblastoma, metaplastic breast cancer, mesothelioma, and sarcomas, both bone and soft tissue (16–21).

ADCs are a tripartite structure consisting of an antibody, a linker, and a payload. Often, these payloads are drugs up to 1,000-fold more toxic than agents used in chemotherapy (22). Therefore, these drugs cannot be used systemically and require coupling to antibodies to direct the cytotoxic effect to specific targets and prevent exposure of healthy tissue. While the clinical efficacy of ADCs is well demonstrated against hematologic and epithelial cancers they have had little success in the context of mesenchymal tumors. Of the monoclonal antibodies (mAb) raised against Endo180 in the original screen (23), one of these, mAb A5/158, was used here to generate an ADC. A5/158 has been extensively characterized (10, 24) and is specific for human Endo180 with its epitope located within CTLD2 (Fig. 1A). Endo180 is rapidly and constitutively recruited into clathrin-coated pits resulting in >50% of the receptor being internalized into intracellular endosomes within 2 minutes (11, 23). Inside the endosomes, the low pH environment results in ligand dissociation and recycling of Endo180 back to the plasma membrane, making it ideal for the rapid uptake of an ADC.

Here, we show high levels of Endo180 protein in the majority of STS of different subtypes, and upregulated *MRC2* expression in multiple sarcoma data sets. Experimentally, an Endo180 ADC demonstrates target-dependent cytotoxicity of multiple sarcoma cell lines *in vitro* and tumor regression and impairment of metastasis in an *in vivo* sarcoma model.

## Materials and Methods

### Reagents and cells

Antibodies and the dilutions used are described in Supplementary Table S1. MG-63, HT-1080, MCF-7, and HT-29 cells were from Isacke laboratory stocks. A-204, G-402, SK-UT-1, and SJSA-1 cells were from Paul Huang (ICR) laboratory stocks. MG-63 cells were transduced with a luciferase2-mCherry vector (mChLuc2). mCherry+ MG-63 cells were enriched by fluorescence-activated cell sorting (FACS). All cells were short tandem repeat tested (StemElite ID System; Promega) and tested negative in routine bimonthly tests for *Mycoplasma* contamination (MycoAlert; Lonza; last tested July 22, 2022). All cell lines were used between 2 and 15 passages after thawing. The generation of anti-human Endo180 mouse mAbs A5/158 and 39.10 has been described previously (23, 24). Antibody concentration was determined by Coomassie Blue or Instant Blue staining of reduced SDS-PAGE gels and interpolation from a standard curve of light chains of either isotype control antibody (BioLegend, 401408) or trastuzumab (Roche, N3031H02).

#### Tissue microarrays (TMA)

All TMAs were constructed as described (25). Details of the rhabdomyosarcoma and adult STS TMAs and associated ethical approvals have been reported previously (26, 27). TMAs had one to five assessable cores per patient sample. The liposarcoma TMAs were constructed with 1-mm diameter cores and comprised cores from tumor biopsies of patients with well-differentiated or dedifferentiated liposarcoma and normal fat samples. Multiple cores were taken from each sample. Where tumor samples contained both well-differentiated and dedifferentiated components, cores from both components were represented on the arrays. Tumor diagnoses had been previously confirmed by a specialist soft-tissue pathologist.

Staining of the TMA sections (4 μm) with the anti-human Endo180 mAb 39.10 was as described previously (24). In brief, slides were incubated with Dako REAL Peroxidase Block (Agilent, S2023) for 5 minutes followed by 39.10 for 1 hour at room temperature. Detection was achieved using Dako Mouse EnVision reagent (Agilent, K4001) for 30 minutes, counterstained using Dako FLEX hematoxylin (Agilent, K8008), and visualized using Dako DAB+ (Agilent, K3468). Positive and negative control samples were included in each run. Stained TMAs were reviewed by a Consultant Pediatric Histopathologist (AK). The staining was both cytoplasmic and perinuclear, and some cases had dot-like accentuation. Cellularity varied depending on sarcoma subtype. Cases with strong 3+ or moderate 2+ immunostaining were scored as positive (majority of tumor cells in all replicate cores staining Endo180-positive) or negative [no or low level (1+) Endo180 staining on tumor cells]. TMA cores with only stromal Endo180 expression were scored negative. TMA cores with equivocal staining or replicate cores with nonconcurrent Endo180 staining were not scored.

### *In vitro* studies

#### Immunofluorescence staining

For the immunofluorescence staining of fixed cells, 2×10^5^ cells were cultured for 2 days on glass coverslips in a 6-cm dish before fixing in 4% paraformaldehyde. Cells were permeabilized in 0.5% Triton X 100 and stained with A5/158 or isotype control antibodies, Alexa dye–labeled phalloidin (Thermo Fisher, A22283) and DAPI (Invitrogen, D1306). For immunofluorescence staining of live cells, 2×10^5^ cells were cultured for 2 days on glass coverslips in a 6-cm dish. A5/158 and isotype control antibodies were conjugated to Alexa Fluor 488 dye (Thermo Fisher, A20181) as per the manufacturer's protocol. Directly conjugated antibodies were diluted in binding buffer (DMEM, 10 mmol/L HEPES pH 7.5, 2 mg/mL BSA; Sigma, A2153) and incubated with cells for 1 hour at 4°C. Coverslips were washed and incubated with LysoTracker Red DND-99 (Thermo Fisher) and Hoechst 3342 nuclear stain (Thermo Fisher) in binding buffer for 30 minutes at 37°C. Cells were washed in binding buffer and fixed in 4% paraformaldehyde. All fluorescence images were collected on a Leica TCS SP8 confocal microscope.

#### Western blotting

Cells were grown to 70% confluence and lysed in complete RIPA buffer (Sigma, R0278) for 30 minutes at 4°C. Lysates were centrifuged at 16,000 × *g* for 30 minutes before the supernatant was removed and sonicated. 15 μg of cell lysate was run on a 4% to 15% PROTEAN TGX gel (Bio-Rad, 456,1083) and transferred using the TransBlot-Turbo transfer system (Bio-Rad). Lysates were subjected to western blotting with the Bio-Rad western blot system. Membranes were blocked with 5% milk for 1 hour before incubation with primary and secondary antibodies.

#### Bioconjugation

Conjugation of A5/158 and isotype control antibodies to saporin was carried out using a streptavidin–biotin conjugation technique previously described (28).

Conjugation of A5/158 and isotype control antibodies to monomethyl auristatin E (MMAE) was carried out by Abzena (Cambridge) Ltd. A5/158 antibody in PBS pH 7.5, 5 mmol/L EDTA (20.7 mg, 1.0 eq.) was reduced by adding 5 mmol/L tris(2-carboxyethyl)phosphine (TCEP, 5.0 eq.). The reduction was allowed to proceed at 40°C for 2 hours with a final antibody concentration of 5 mg/mL. The reduction mixture was allowed to cool to 22°C, and a 10 mmol/L solution of mc-vc-PAB-MMAE in DMSO (380 μL, 9.0 eq.) was added (final concentration of 10% DMSO and antibody concentration of 4.0 mg/mL). The conjugation reaction was allowed to proceed at 22°C for 2 hours. The conjugate was purified by ultrafiltration/diafiltration (UF/DF) using a Vivaspin 20 (30 kDa MWCO) and buffer exchanged into PBS.

Isotype antibody in PBS pH 7.5, 5 mmol/L EDTA (20 mg, 1.0 eq.) was reduced as described above. A 7.6 mmol/L solution of mc-vc-PAB-MMAE in DMSO (105 μL, 6.0 eq.) was added to the reduced antibody (final concentration of 10% DMSO and antibody concentration of 4.0 mg/mL). The conjugation reaction and antibody purification were as described above.

To determine the drug-to-antibody ratio (DAR), the ADCs were analyzed by hydrophobic interaction chromatography (HIC) using a Tosoh TSKgel Butyl-NPR column. The resulting ADCs had a DAR of 4.0 and 3.4 for A5/158-vc-MMAE and Isotype-vc-MMAE, respectively. Aggregation of conjugates was determined by size exclusion chromatography (SEC) using a Waters BEH 200 Å column.

#### ADC cell proliferation/viability assays

1×10^3^ to 2×10^3^ cells/well were seeded into a 96-well plate. After 48 hours, cells were treated with antibodies/ADCs. Cell growth was tracked and quantified by the Live-Cell Analysis System IncuCyte (EssenBioscience). At the endpoint, cell viability was quantified by CellTiter-Glo (Promega).

### *In vivo* studies

All animal work was carried out under UK Home Office Project license P6AB1448A (Establishment License, X702B0E74), which outlines experimental protocols and endpoints. Work was approved by the Animal Welfare and Ethical Review Body at the ICR. Six- to 7-week-old female NOD.Cg-*Prkdc^scid^ Il2rg^tm1W^^j^^l^*/SzJ (NSG) mice were purchased from Charles River. Animals were housed in IVC-type cages, which are run under negative airflow. Mice had food and water ad libitum and were monitored daily by the ICR Biological Services Unit staff.

#### Subcutaneous inoculation

1×10^6^ MG-63-mChLuc2 cells were injected subcutaneously into the flank under general anesthesia. Tumor growth was measured 3 times a week using calipers. Tumor volumes and growth rates were calculated as described previously (12).

#### ADC treatment

Antibodies and ADCs were diluted in PBS immediately prior to administration. Vehicle (PBS) or 10 mg/kg of A5/158, isotype-vc-MMAE or A5/158-vc-MMAE were administered into the lateral tail vein of mice twice a week for 2 weeks. Primary tumors were weighed at necropsy.

#### IVIS imaging

Mice were injected intraperitoneally with 150 mg/kg D-luciferin (Caliper Life Sciences), and after 5 minutes mice were imaged *in vivo* using an IVIS imaging chamber (IVIS Illumina II). Organs were also imaged individually *ex vivo*. Luminescence measurements (photons/second/cm^2^) were acquired over 1 to 60 seconds and analyzed using the Living Image software (PerkinElmer) using a constant-sized region of interest over the tissues.

#### IHC

Mouse tumors and organs were harvested at necropsy, formalin-fixed and paraffin-embedded. Tumor sections were stained with human Endo180 mAb 39.10 as described above for TMA staining. For lungs and livers, 3 sections approximately 150 μm apart were stained for human lamin A/C. Images of stained sections were acquired on the NanoZoomer Digital Pathology (Hamamatsu). Quantification of the number and size of lamin A/C-positive lesions in lungs and livers was carried out using QuPath v0.3.0 (RRID:SCR_018257; ref. 29). Briefly, a pixel classifier was trained for lamin A/C-positive cell recognition using the artificial neural network algorithm. Annotations were drawn around whole tissue sections which were divided into tiles using SLIC superpixel segmentation. Identified by the trained pixel classifier, tumor lesions were quantified by converting tiles to annotations. Metastatic lesions were defined as > 1,500 μm^2^ (approximately >10 tumor cells).

#### Real-time quantitative polymerase chain reaction (RT-qPCR)

Tumor tissue was harvested at necropsy and snap-frozen in liquid nitrogen. Tumor tissue was homogenized in Precellys Lysing Kit tubes (P000911-LYSK0-A) containing RLT buffer (QIAGEN) plus 1:100 β-mercaptoethanol. RNA was isolated using the RNeasy Mini Kit (QIAGEN, 74104) and cDNA was generated using the Superscript kit (Invitrogen, 18091050) according to the manufacturer's instructions. RT-qPCR was performed using TaqMan Gene-Expression Assay Probes (Life Technologies; UBC probe, Hs00824723_m1; MRC2, Hs00977846_m1) on a QuantStudio 6 Flex Real-Time PCR system (Applied Biosystems) and relative quantification was performed using QuantStudio Real-Time PCR software. Each reaction was performed in triplicate. Relative *MRC2* expression levels were normalized to *UBC*.

### Statistical analysis

Statistical analysis was performed using GraphPad Prism 9 (RRID:SCR_002798). Data were normality tested before one-way ANOVA analysis was performed with the Tukey test for multiple comparison of parametric data, or Kruskal–Wallis test was performed with Dunn multiple comparison test for nonparametric data. Nonsignificant (ns) *P* values > 0.05.

### Data availability

Series matrix files of whole transcript expression data from STS tumors and control normal fat specimens were downloaded from the Gene-Expression Omnibus (GEO) site: GSE21122 (RRID:SCR_005012; ref. 30). Gene expression of *MRC2* from the Innovative Therapies for Children with Cancer (ITCC) and The Cancer Genome Atlas (TCGA) data sets was retrieved using the R2: Genomics Analysis and Visualization Platform (http://r2.amc.nl and http://r2platform.com). *MRC2* gene expression from Cancer Cell Line Encyclopedia (CCLE) data sets was retrieved from https://depmap.org/portal/ (RRID:SCR_017655). Expression of *MRC2* in pediatric sarcoma and normal tissue was retrieved from the National Cancer Institute (NCI) OncoGenomics data portal https://clinomics.ccr.cancer.gov/clinomics/public/.

## Results

### Endo180 is expressed on multiple sarcoma subtypes

Endo180 protein expression was assessed by IHC using the anti-Endo180 mAb 39.10 (Fig. 1A) in rhabdomyosarcoma, liposarcoma, leiomyosarcoma, synovial sarcoma, fibrosarcoma, and undifferentiated pleomorphic sarcoma (UPS) TMAs (Fig. 1B and C; Supplementary Fig. S1). We have previously reported the specificity of the 39.10 antibody (24). High levels of Endo180 protein are detected in the majority of STS: 64.0% of rhabdomyosarcomas (126/197), 38.5% well-differentiated liposarcomas (20/52), 93.1% dedifferentiated liposarcomas (54/58), 64.2% leiomyosarcomas (97/151), 76.1% synovial sarcomas (32/42), 87.5% fibrosarcomas (21/24), and 64.3% of UPS (65/101; Fig. 1C). Examples of cores scored positive and negative are provided in Supplementary Fig. S1. Of note, due to its constitutive recycling properties, Endo180 staining is frequently observed in the perinuclear region characteristic of clustered intracellular endosomes (11). In samples with Endo180-negative tumor cells, Endo180-positive CAFs are frequently detected in the tumor stroma (Supplementary Fig. S1). Fibroblasts with low-level Endo180 expression are detected in normal human tissue controls (Supplementary Fig. S2A).

Equivalent findings were obtained by examining *MRC2* (Endo180) gene expression in STS of different subtypes from Barretina and colleagues (30), with *MRC2* highly expressed in the majority of adult STS compared with normal fat (Fig. 1D). A similar profile is seen in the STS samples from the TCGA database (Supplementary Fig. S2B). Within pediatric data sets, a comparison of cancer samples and normal tissue from the NCI OncoGenomics database (Supplementary Fig. S2C) also demonstrates higher *MRC2* expression in the majority of sarcoma samples compared with the mean normal tissue expression (Supplementary Fig. S2c), with notable elevation of *MRC2* expression in osteosarcomas. An equivalent expression pattern has been reported in samples from the St Jude PeCan Data Portal (21). Finally, gene expression in rhabdomyosarcomas from the ITCC data sets (Fig. 1E) shows increased *MRC2* expression compared with normal skeletal muscle. There is no significant difference between the rhabdomyosarcoma subtypes, categorized by fusion status of forkhead (*FKHR*) to paired box 3 (*PAX3*) or PAX 7. Together, IHC staining and bioinformatic analysis demonstrate that expression of Endo180 in sarcomas and CAFs is elevated compared with normal tissue fibroblasts, indicating that on-target, off-tumor toxicities would be limited in patients.

### A5/158 recognizes Endo180 expressed on sarcoma cell lines

**Figure 2.**
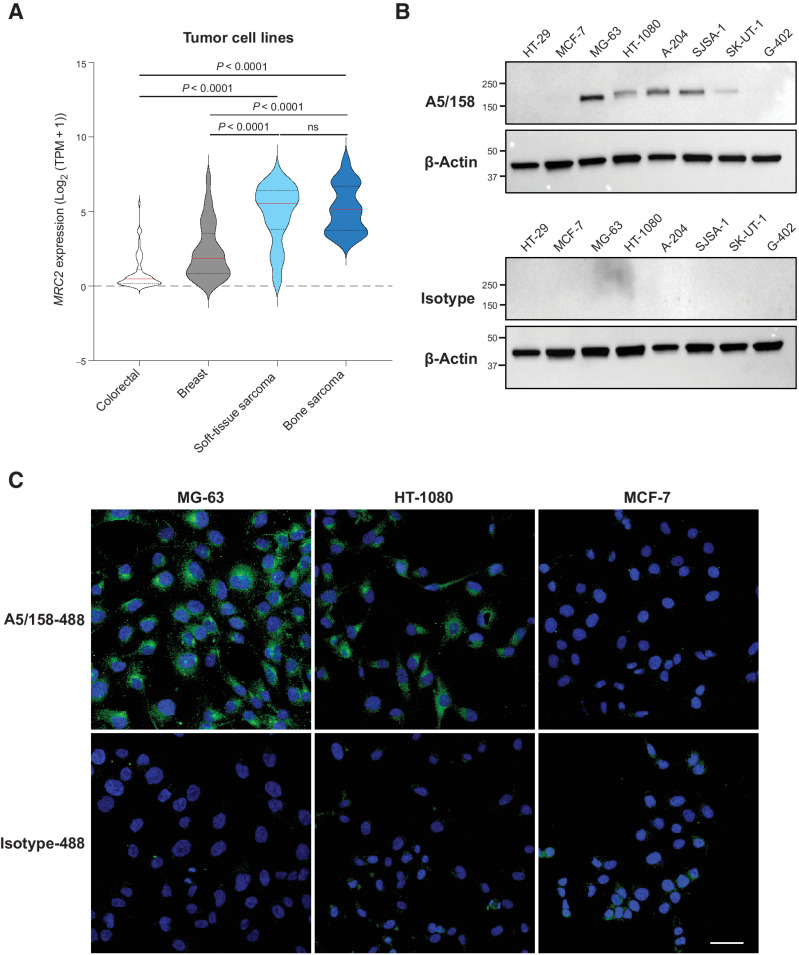
A5/158 recognizes Endo180 expressed on sarcoma cell lines. **A,** Endo180 (*MRC2*) gene expression from CCLE in colorectal (*n* = 70), breast cancer (*n* = 61), STS (*n* = 60), and bone sarcoma (*n* = 39) cell lines (violin plots, red lines indicate median expression levels, dotted lines indicate upper and lower quartiles, Kruskal–Wallis test, Dunn multiple comparison test). **B,** Western blot analysis of Endo180 in epithelial tumor (HT-29 and MCF-7) and sarcoma (MG-63, HT-1080, A-204, SJSA-1, SK-UT-1, and G-402) cell lines. Membranes were probed with anti-Endo180 mAb A5/158 (top) or isotype control antibody (bottom). Molecular size markers are in kDa. **C,** Confocal images of sarcoma cell lines (MG-63 and HT-1080) and breast cancer cells (MCF-7) stained A5/158 or isotype control antibody directly conjugated to Alexa Fluor 488 (green). Nuclei were counterstained with DAPI (blue). Representative images out of two fields of view from experiments repeated on at least 3 occasions with equivalent findings. Scale bar, 50 μm.

Analysis of expression data from the CCLE is consistent with the IHC staining and gene-expression analysis of primary tumors (Fig. 1; Supplementary Fig. S1), showing significantly higher *MRC2* expression in both STS and bone sarcoma cell lines compared with breast or colorectal cancer cell lines (Fig. 2A). To evaluate if the anti-Endo180 mAb A5/158, which recognizes an epitope in CTLD2 (Fig. 1A; ref. 31), is a suitable candidate for development into an ADC, we assessed its binding specificity in multiple sarcoma cell lines. Sarcoma cell lines MG-63 (osteosarcoma), HT-1080 (fibrosarcoma), A-204 (malignant rhabdoid tumor), SJSA-1 (osteosarcoma), SK-UT-1 (uterine leiomyosarcoma), and G-402 (leiomyoblastoma), along with the known Endo180-negative epithelial cell lines HT-29 (colorectal cancer) and MCF-7 (breast cancer), were subjected to western blotting with A5/158 or an isotype control antibody (Fig. 2B). As previously reported (23, 24), Endo180 protein is not detected in either epithelial cancer cell line and, consistent with the CCLE data (Fig. 2A), the majority of sarcoma cell lines show Endo180 protein expression detected by A5/158 but not isotype control antibody (Fig. 2B). Lower Endo180 protein levels in the SK-UT-1 and G-402 cell lines reflects lower *MRC2* expression in the CCLE data set compared with the other sarcoma cell lines.

In the immunofluorescence staining of permeabilized cells in culture using A5/158 directly conjugated to an Alexa Fluor 488 dye (A5/158-488), both the MG-63 and HT-1080 sarcoma cell lines show intracellular vesicular staining, clustered in the perinuclear region (Fig. 2C) and mirroring the staining pattern in the STS TMAs (Fig. 1B; Supplementary Fig. S1). In contrast, no staining is detected in the MCF-7 breast cancer cells or with the isotype control antibody. Examining a broader range of sarcoma cell lines (Supplementary Fig. S3a), levels of immunofluorescence staining concur with the CCLE expression (Fig. 2A) and western blotting (Fig. 2B) data and additionally demonstrate that Endo180 expression is homogeneous in the majority of cell lines, with only slight heterogeneity in staining intensity seen in SK-UT-1 and G-402 lines.

### A5/158 is suitable for intracellular drug delivery

**Figure 3.**
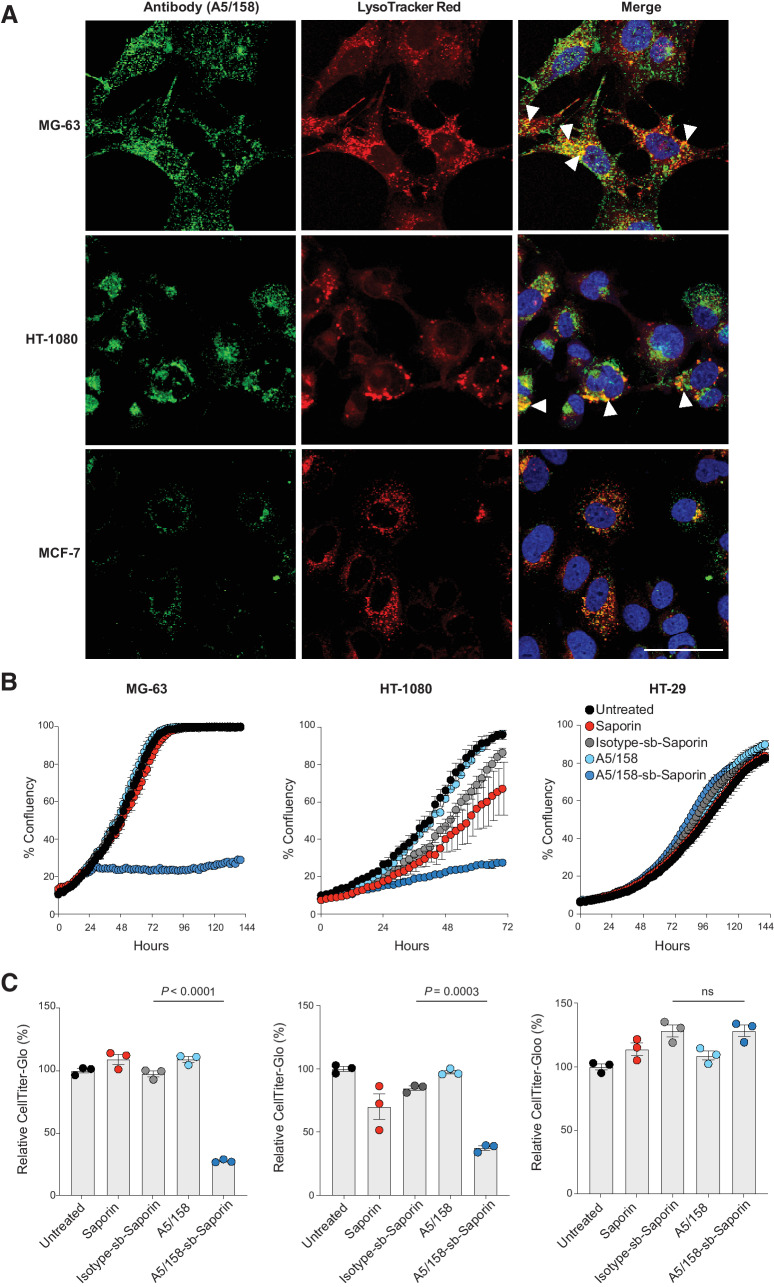
A5/158 is suitable for intracellular drug delivery. **A,** Confocal images of sarcoma and epithelial cells cultured with A5/158-488 (green) for 1 hour at 4°C followed by incubation with LysoTracker Red (red) and Hoechst 33342 (blue) for 30 minutes at 37°C. White arrowheads indicate areas of green–red colocalization. Representative images out of two fields of view, from experiments repeated on at least 3 occasions with equivalent findings. Scale bar, 50 μm. **B,** Representative graphs of MG-63, HT-1080, and HT-29 cell growth (independent experiments; MG-63, *n* = 3; HT-1080, *n* = 2; HT-29, *n* = 1) treated with A5/158 alone, A5/158-sb-saporin, Isotype-sb-saporin, or saporin alone. MG-63 and HT-29 were cultured with 0.5 μg/mL of each treatment or molar equivalent of saporin alone for 144 hours. HT-1080 was cultured with 1.5 μg/mL of each treatment or molar equivalent of saporin alone for 72 hours (*n* = 3 wells per condition; mean values ±SEM). Cell growth measured by confluency on the IncuCyte. **C,** At endpoint, cell viability readouts were determined by CellTiter-Glo. Data are normalized to untreated cells (*n* = 3 wells per condition, mean values ± SEM).

Endo180 is known to be rapidly and constitutively internalized into endosomes where ligand detaches in the low pH environment and is trafficked to the lysosomes for degradation, whereas the receptor is recycled back to the plasma membrane (10, 11, 23). To assess whether the internalizing receptor can deliver an intact antibody intracellularly, A5/158-488 or Isotype-488 was incubated with unfixed Endo180-positive MG-63 and HT-1080, and Endo180-negative MCF-7 cells for 1 hour at 4°C before unbound antibody was removed by washing and coverslips were incubated with the lysosomal marker LysoTracker Red and Hoechst nuclear stain for a further 30 minutes at 37°C. Strong punctate fluorescence in the green channel is detected in MG-63 and HT-1080 sarcoma cells (Fig. 3A, left) demonstrating A5/158 has been internalized. No A5/158-488 fluorescence is detected in the MCF-7 cells. Additionally, no fluorescence is detected with the Isotype-488 antibody (Supplementary Fig. S3b). Merged images of internalized A5/158-488 and the LysoTracker highlight areas of colocalization, indicating that A5/158 is trafficked to the lysosomes (Fig. 3A, right).

To confirm that internalized antibodies are capable of delivering a cytotoxic drug intracellularly, A5/158 was conjugated to the ribosome inactivating toxin, saporin, using a streptavidin–biotin (sb) rapid conjugation technique (28). MG-63, HT-1080, and MCF-7 cells incubated with unconjugated A5/158 have no impairment in cell growth or cell viability, determined at endpoint by CellTiter-Glo (Fig. 3B and C). Saporin is not membrane permeable and therefore not able to cause cell cytotoxicity alone. However, when conjugated to A5/158, MG-63 and HT-1080 cell growth and cell viability are significantly impaired. As saporin is conjugated via a noncleavable linker, these data indicate that A5/158-sb-Saporin is internalized into Endo180 expressing cells, trafficked to the lysosome for degradation and the toxin released intracellularly. This effect is not seen in MCF-7 cells or with Isotype-sb-Saporin conjugate treatment demonstrating that inhibition of cell growth and viability is dependent on Endo180-mediated A5/158-sb-Saporin internalization.

### Endo180 expressing cell lines are sensitive to A5/158-vc-MMAE

**Figure 4.**
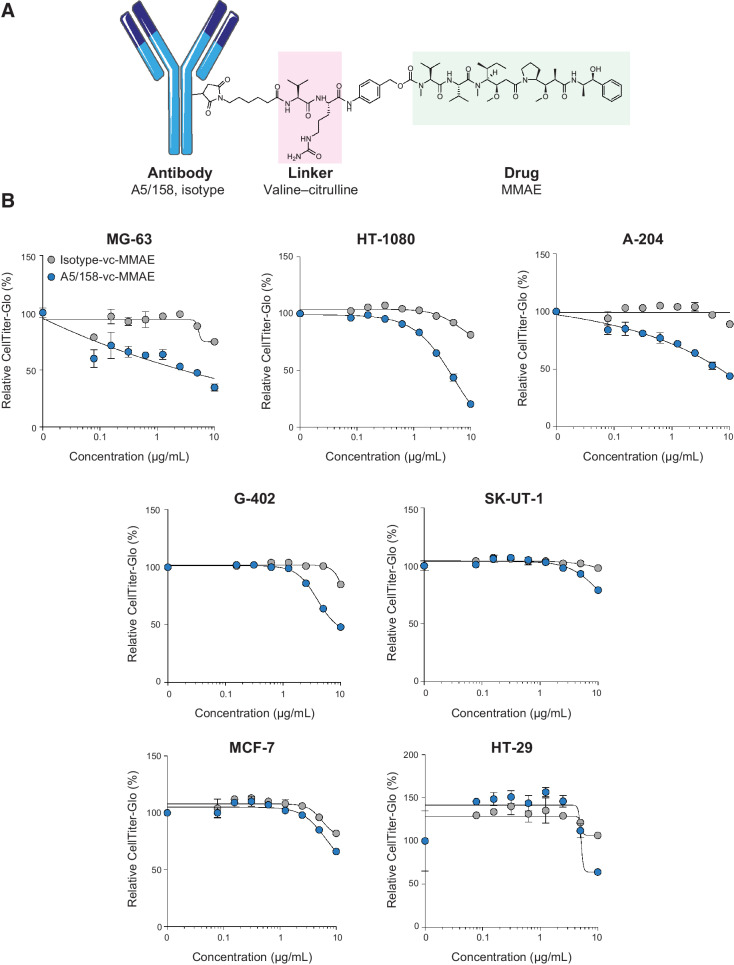
Endo180 expressing cell lines are sensitive to A5/158-vc-MMAE. **A,** Schematic representation of an antibody conjugated to an MMAE payload (green) via a cleavable valine–citrulline linker (pink). **B,** Endo180-positive and Endo180-negative cell lines were treated with A5/158-vc-MMAE or isotype-vc-MMAE for 72 hours. Shown are representative graphs (independent experiments *n* = 3) of cell viability readouts determined by CellTiter-Glo. Data are normalized to untreated cells (*n* = 3 wells per condition, mean values ± SEM).

Next, an ADC was created by conjugating A5/158 to the microtubule targeting drug monomethyl auristatin E (MMAE) via a dipeptide valine–citrulline linker (vc) which, upon internalization, is cleaved by lysosomal cathepsin enzymes (Fig. 4A). This linker–payload combination was selected as it is utilized by 4 of the 11 currently FDA-approved ADCs, and in 5 of the 12 ADCs being developed for the treatment of sarcoma (8). In addition, the membrane permeability of MMAE can cause a desirable bystander killing effect on the surrounding cells. The isotype control antibody was conjugated to the same linker–payload combination, and the resulting ADCs were termed A5/158-vc-MMAE and Isotype-vc-MMAE. HIC was used to determine the composition of the DAR for each ADC (Supplementary Fig. S4a). The average DAR achieved was 4.0 and 3.4 for A5/158-vc-MMAE and Isotype-vc-MMAE, respectively, comparable with the average DAR of 3.5 of the ADC Trastuzumab-DM1, which is approved for the treatment of HER2-positive breast cancers. Conjugation of A5/158 to MMAE does not increase antibody aggregation, assessed by SEC (Supplementary Fig. S4b), nor disrupt its ability to bind Endo180, assessed by western blotting (Supplementary Fig. S4c).

To examine the efficacy of A5/158-vc-MMAE *in vitro*, increasing concentrations of A5/158-vc-MMAE and Isotype-vc-MMAE were incubated with Endo180-positive sarcoma (MG-63, HT-1080, and A-204), Endo180-low sarcoma (G-402 and SK-UT-1), and Endo180-negative (MCF-7 and HT-29) control cell lines (Fig. 4B). Endo180-positive cell lines are sensitive to A5/158-vc-MMAE while Endo180-negative cell lines are comparatively resistant. Although to a lesser extent than Endo180-positive cell lines, Endo180-low cell lines still show a specific decrease in viability when treated with A5/158-vc-MMAE, demonstrating a correlation between Endo180 expression and sensitivity to A5/158-vc-MMAE.

### A5/158-vc-MMAE decreases sarcoma tumor growth and metastasis

As MG-63 cells show sensitivity to A5/158-vc-MMAE *in vitro* (Fig. 4B), a sarcoma tumor model using these cells was established subcutaneously in immunodeficient NSG mice to assess efficacy *in vivo*. In a pilot tolerability experiment (Supplementary Fig. S5a), mice were treated with 2.5, 5, and 10 mg/kg A5/158-vc-MMAE or vehicle (PBS) when MG-63-mChLuc2 tumors were ∼3.5 mm in diameter (day 10). Alternatively, two mice were treated with vehicle until tumors were ∼8.5 mm in diameter (day 24) and then treated with two doses of 5 mg/kg A5/158-vc-MMAE. The pilot experiment was terminated on day 32 due to the development of large lymph node metastases in the vehicle-treated mice. No mice treated with A5/158-vc-MMAE showed any clinical signs of distress or weight loss, indicating that the ADC does not cause dose-limiting toxicities (Supplementary Fig. S5b). A dose-dependent inhibition of tumor growth is observed in mice treated with A5/158-vc-MMAE, with almost complete tumor regression in the mouse treated with 4 doses 10 mg/kg of A5/158-vc-MMAE (Supplementary Fig. S5c-S5f). 39.10 staining of tumors at necropsy indicates that surviving tumor cells retain Endo180 expression *in vivo*, indicating that tumors would be sensitive to further ADC treatment (Supplementary Fig. S5f). Examination of organs at necropsy and IHC staining of human lamin A/C used to identify MG-63 tumor cells demonstrates all concentrations of A5/158-vc-MMAE, including 5 mg/kg treatment starting at day 24, impair metastatic colonization of the lungs and liver (Supplementary Fig. S6).

**Figure 5.**
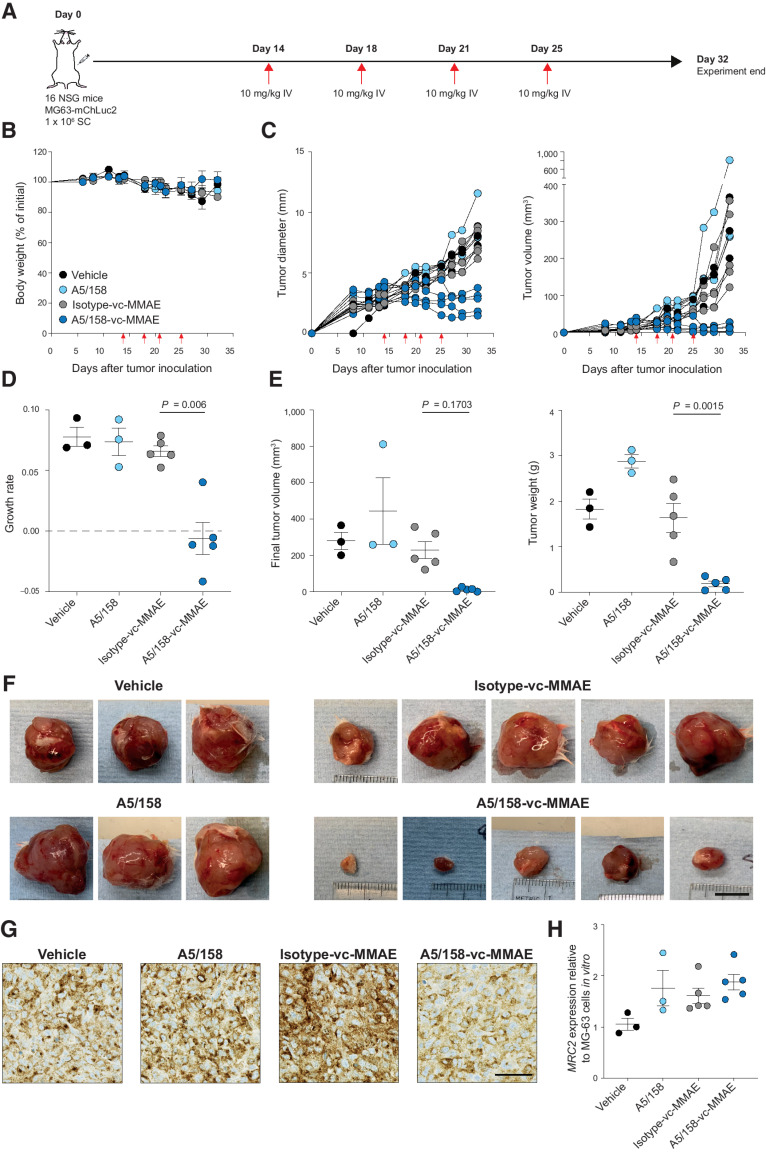
A5/158-vc-MMAE impairs sarcoma tumor growth. **A,** Experimental schematic. 1×10^6^ MG-63-mChLuc2 cells were injected subcutaneously (s.c.) into the flank of NSG mice. On day 14, once tumors reached ∼3 mm in diameter, mice were randomized and began treatment. Mice were treated intravenously (i.v.) with 10 mg/kg of A5/158, Isotype-vc-MMAE, A5/158-vc-MMAE, or vehicle (PBS) twice a week for a total of 4 doses (vehicle, *n* = 3; A5/158, *n* = 3; isotype-vc-MMAE, *n* = 5; A5/158-vc-MMAE, *n* = 5; red arrows indicate treatment days). **B,** Bodyweight of treated mice relative to weight on day 0. **C,** Tumor growth in individual mice measured in tumor diameter and volume. **D,** Growth rate of tumors (one-way ANOVA, Tukey multiple comparison test). Data shown are mean values ±SEM. **E,** Final tumor volumes (Kruskal-Wallis, Dunn multiple comparison test) and weights (one-way ANOVA, Tukey multiple comparison test). All are shown as mean values ± SEM. **F,***Ex vivo* images of MG-63 tumors. Scale bar, 10 mm. **G,** Representative IHC images of MG-63 tumors stained for Endo180. Scale bar, 50 μm. **H,** Relative Endo180 (*MRC2*) expression of MG-63 tumors after treatment in comparison with parental MG-63 cells *in vitro,* analyzed by RT-qPCR and normalized to *UBC* endogenous control. Data shown are mean values ± SEM.

Based on the pilot tolerability study, NSG mice were inoculated subcutaneously with 1×10^6^ MG-63-mChLuc2 cells. Once the tumors had reached ∼3 mm in diameter, mice were administered intravenously with vehicle (PBS), A5/158 alone, A5/158-vc-MMAE, or Isotype-vc-MMAE (all 10 mg/kg) twice weekly for a total of four doses (Fig. 5A). No toxicity is observed from any treatment through either monitoring animal bodyweight (Fig. 5B) or clinical signs of distress. No effect on tumor growth rate nor final tumor size or weight is observed with A5/158 alone or Isotype-vc-MMAE treatment compared with vehicle-treated mice. By contrast, treatment with A5/158-vc-MMAE results in tumor regression (Fig. 5C–F). In line with the pilot tolerability study (Supplementary Fig. S5f), the surviving cells in the A5/158-vc-MMAE–treated MG-63 tumors retain Endo180 expression as monitored by IHC and RT-qPCR (Fig. 5G and H).

**Figure 6.**
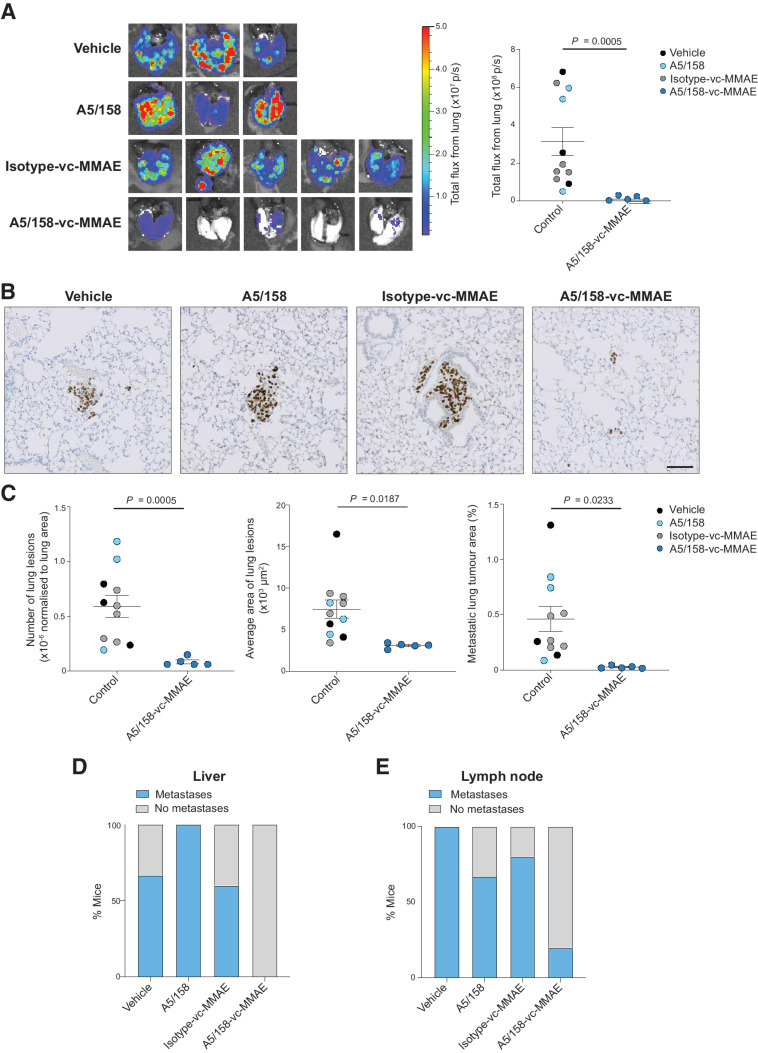
A5/158-vc-MMAE decreases the number and size of spontaneous metastases. Continuation of Fig. 5. **A,***Ex vivo* IVIS imaging and quantification of lungs at endpoint (mean values ± SEM, two-sided Mann–Whitney *U* test). **B,** Representative FFPE lung sections stained for lamin A/C at 3 levels 150 μm apart. Scale bar, 100 μm. **C,** Quantification of the number of metastatic lung lesions, average area of lung lesions, and % metastatic area of lung lesions (mean of three sections per mouse). Data shown are mean values ± SEM; number of lung lesions, two-sided Mann–Whitney *U* test; average area of lung lesions and metastatic tumor area, two-sided unpaired *t* test. **D,** Proportion of mice with liver metastases. **E,** Proportion of mice with lymph node metastases.

*Ex vivo* IVIS imaging of the lungs of the animals from Fig. 5 shows a significant reduction in bioluminescent signal in the lungs of A5/158-vc-MMAE—treated mice compared with control mice (Fig. 6A). Human lamin A/C staining reveals a substantial reduction in both the number and size of lung metastases in A5/158-vc-MMAE–treated mice (Fig. 6B and C). Similarly, 8 of 11 control mice developed liver metastases in contrast to 0 of 5 mice treated with A5/158-vc-MMAE (Fig. 6D; Supplementary Fig. S7). Finally, 9 of 11 control mice, but only 1 of 5 A5/158-vc-MMAE–treated mice, developed overt lymph node metastases (Fig. 6E). Taken together, these data demonstrate that treatment of MG-63 tumor-bearing mice with A5/158-vc-MMAE effectively induces primary tumor regression and inhibits the outgrowth of metastatic lesions in the lung, liver, and lymph nodes.

## Discussion

At the time of writing, there are 11 FDA-approved ADCs available in the clinic, 7 of which have only been approved since 2018, making ADCs one of the most rapidly growing fields of cancer therapeutics. Owing to the variable responses of sarcoma tumors to first-line therapies, research into the development of personalized medicines has increased. Approaches have included targeting antigens expressed on sarcomas with ADCs, such leucine-rich repeat containing 15 (LRRC15; ref. 32), glycoprotein nonmetastatic b protein (GPNMB; ref. 33), and neural cell adhesion molecule (NCAM, CD56; ref. 34) plus a membrane type 1–matrix metalloproteinase (MT1-MMP) bicycle toxin conjugate (21). In total, 9 immunoconjugates targeting sarcoma have entered clinical trials (8). Despite these advancements, no antibody-based therapies have yet been approved for sarcoma treatment and, therefore, there remains a significant unmet clinical need for patients, especially in metastatic settings.

Here, we report the first *in vivo* assessment of an Endo180 (*MRC2*)-directed ADC targeting in sarcoma. High levels of Endo180 protein have been reported previously in osteosarcomas (17, 19, 21), with Endo180 identified as one of the most upregulated genes in proteomic profiling, transcriptomic analysis, and IHC staining of osteosarcoma patient samples and cell lines, compared with normal tissues and other pediatric cancers (21). Despite this, Endo180 expression in the broader range of sarcoma subtypes has not been investigated. In this study, we assessed Endo180 protein levels in 625 STS clinical samples and complemented this with bioinformatics analysis of sarcoma gene-expression data sets. Strikingly, high levels of Endo180 protein are detected in the majority (415 of 625) of the common STS subtype tumors. A similar picture emerges assessing *MRC2* expression in sarcoma gene-expression profiling data sets, highlighting the potential of Endo180 as a therapeutic target.

In this study, we show that an A5/158-vc-MMAE ADC specifically kills Endo180-expressing sarcoma cell lines. This is in agreement with a previous study showing that an alternative Endo180 mAb vc-MMAE conjugate reduced viability of Endo180-positive sarcoma cell lines *in vitro* and had efficacy against an Endo180-positive human myeloid leukemia xenograft (19). Similarly, it has been demonstrated recently that Endo180 expression is upregulated on malignant mesotheliomas and that an Endo180-targeting ADC causes the cytotoxicity of mesothelioma cell lines *in vitro* (16).

Previously, we demonstrated that an anti-Endo180 ^125^I-Fab' fragment is rapidly internalized from the cell surface and that ∼70% of internalized Fab' is degraded intracellularly without affecting the half-life of Endo180 receptor, as measured in a ^35^S-methionine pulse-chase assay (23). This early study provided two important pieces of information. First, an Endo180-targeting ADC would be able to release its cytotoxic payload intracellularly, and indeed, as demonstrated here, the A5/158-vc-MMAE is trafficked into the lysosomes for degradation. Second, the target receptor would remain present on any cell surviving an initial encounter with the ADC, as observed in Fig. 5G and H, and hence would be sensitive to further rounds of treatment. This is important as the development of resistance to ADCs utilizing the vc-MMAE linker-payload complex has been observed and attributed to the downregulation of the target receptor and/or removal of MMAE by the efflux pump multidrug resistance protein 1 (MDR1; refs. 35–37). Approaches such as modification of the ADC linker, conjugation with drugs that are not MDR1 substrates, or engineering dual-drug ADCs have been developed to maintain efficacy in MDR1-expressing tumors (37–39). Consequently, should resistance to MMAE occur via MDR1-mediated efflux, such modified ADCs could restore antitumor activity as retention of Endo180 expression would ensure that a second ADC is still efficiently internalized.

The first limitation of this study, and previous Endo180 ADC studies (16, 19), is the use of an ADC specific for human Endo180 requiring human tumors to be grown in immunodeficient mice. This precludes an assessment of on-target off-tumor toxicities and immune engagement. In the model used here, no adverse effects were seen in the body weight or other signs of ill health in the mice treated with either the A5/158-vc-MMAE or Isotype-vc-MMAE ADCs, demonstrating the stability of the ADC in the circulation and the absence of off-target toxicities. Although the Endo180 knockout mouse has no discernible abnormalities, Endo180 is expressed in normal tissue fibroblasts, albeit at a lower level than in sarcomas (Fig. 1; Supplementary Figs. S1 and S2). Consequently, further studies are required to determine if there are any on-target, off-tumor toxicities associated with this therapy. Immune recruitment by antibodies has been implicated with antitumor efficacy via antibody-dependent cellular cytotoxicity and antibody-dependent cellular phagocytosis (ADCP) carried out by natural killer and macrophage cells, respectively. This ADCC effect has been attributed to the HER2-targeting antibody, trastuzumab, in preclinical models and likely contributes to the efficacy of the corresponding ADC, trastuzumab-DM1 (T-DM1; refs. 40–42), although this would not be evident in an immunodeficient setting. Finally, in addition to expression on the sarcoma tumor cells, Endo180 levels are also elevated in CAFs compared with normal tissue fibroblasts (13). Due to the species specificity of A5/158-vc-MMAE, the effects of targeting both Endo180-positive human tumor cells and mouse CAFs could not be assessed. The development of an ADC recognizing mouse Endo180 will be required to perform a full preclinical assessment of any normal tissue toxicity, to examine the immune engagement of the ADC, which could enhance the antitumor effect of the therapy at both the primary and metastatic sites and to determine the benefit of targeting both tumor cells and stromal CAFs.

The second limitation of this study is the potency of the A5/158-vc-MMAE ADC, which has an IC_50_ in Endo180-expressing cells of ∼5 μg/mL, making it less effective than other ADCs with an MMAE payload (19, 43, 44). However, in these other studies, it is notable that the control ADCs were substantially more cytotoxic likely reflecting differences in target cell density and/or assay duration. Nonetheless, the A5/158 ADC requires further optimization to improve its efficacy prior to further clinical development. Although higher DARs can increase the cytotoxicity of an ADC, it will also be particularly important to investigate the efficacy and optimal dosing/scheduling of A5/158 conjugated to alternative payloads such as SN-38, duocarmycin, pyrrolobenzodiazepine (PBD) dimers, and exatecan, which have been used in the development of more recent ADCs against sarcomas and other tumor types (45–49). Additionally, in mice treated with A5/158-vc-MMAE, not all Endo180-positive MG-63 tumor cells were depleted (Fig. 5G and H), and although this may reflect the ADC potency, lack of penetration of the ADC into the tumor may also play a role. The use of an antibody fragment or alternative scaffold drug conjugates may improve tumor penetration owing to their smaller size; however, these small format drug conjugates are often associated with more rapid clearance and therefore the duration of treatment can be limited (50).

In summary, the A5/158-vc-MMAE ADC specifically kills Endo180-expressing cell lines, causes regression of an Endo180-positive osteosarcoma tumor model, and reduces metastatic colonization of the lung, liver, and lymph nodes. This identifies Endo180 as a promising pharmacologic target for the treatment of sarcoma, particularly of metastatic sarcomas for which there are currently no specific and effective therapies.

## Supplementary Material

Supplementary Table S1 Antibodies and their dilutions

Supplementary Figure S1 Examples of Endo180 positive and negative staining in subtypes of soft tissue sarcoma tumor cores.

Supplementary Figure S3 Endo180 protein expression in sarcoma cell lines compared to epithelial cancer cells by immunofluorescence. Additionally, this figure shows the isotype control antibody conjugated to AlexaFluor 488 is not internalized into Endo180 expressing sarcoma cell lines and is not trafficked to the lysosome.

Supplementary Figure S2 Endo180 protein and gene expression in healthy human tissue compared to sarcoma subtypes.

Supplementary Figure S4 Composition and drug to antibody ratio of A5/158 and the isotype control antibody conjugated to vc-MMAE. This figure additionally shows that that conjugation of A5/158 and the isotype control antibody to vc-MMAE does not induce antibody aggregation or disrupt Endo180 protein recognition.

Supplementary Figure S5 In vivo pilot experiment of NSG mice bearing MG-63 subcutaneous tumours treated intravenously with different concentrations of A5/158-vc-MMAE.

Supplementary Figure S6 Continuation of Figure S5 showing the effect of A5/158-vc-MMAE treatment on the spontaneous metastatic spread of MG-63 cells to the lungs and liver.

Supplementary Figure S7 Immunohistochemical staining and quantification of the effect of A5/158, A5/158-vc-MMAE and Isotype-vc-MMAE treatment on the spontaneous metastasis of MG-63 cells to the liver in NSG mice.
