# Supplementary material for: Endo180 (MRC2) Antibody–Drug Conjugate for the Treatment of Sarcoma
Source: Mol Cancer Ther. 2022 Nov 18;22(2):240–53. doi: 10.1158/1535-7163.MCT-22-0312 (PMC9890142; doi:10.1158/1535-7163.MCT-22-0312)
Supplement: Supplementary Table S1 — Antibodies and their dilutions [file mct-22-0312_supplementary_table_s1_suppst1.pdf]

Supplementary Table S1. Antibodies

Antibody (Clone)	Species	Source (Catalogue number)	Application	Dilution/ Concentration used (µg/mL)
Endo180 (39.10)	Mouse	Isacke lab	IHC	22
Endo180 (A5/158)	Mouse IgG_1, κ	Isacke lab	WB<br>IF	0.7<br>5
Endo180 (A5/158) - Alexa488	Mouse	Isacke lab	IF	5
Isotype control	Mouse IgG_1, κ	BioLegend (401408)	WB	0.7
Isotype control - Alexa488	Mouse	BioLegend (401408)	IF	5
β-actin	Mouse	Sigma (A5441)	WB	1:10,000
Lamin A/C (human)	Rabbit	Abcam (ab108595	IHC	1:750
Alexa Fluor 488-anti-mouse IgG	Goat	Molecular Probes (A11001)	IF	1:1,000
Anti-mouse IgG (HRP)	Goat	Abcam (ab205719)	WB	1:5,000
Anti-rabbit IgG (HRP)	Goat	Abcam (ab205718)	IHC	1:10,000

IF, immunofluorescence; IHC, immunohistochemistry; WB, western blotting
