# Supplementary material for: Endo180 (MRC2) Antibody–Drug Conjugate for the Treatment of Sarcoma
Source: Mol Cancer Ther. 2022 Nov 18;22(2):240–53. doi: 10.1158/1535-7163.MCT-22-0312 (PMC9890142; doi:10.1158/1535-7163.MCT-22-0312)

Supplementary Figure S1. Immunohistochemical Endo180 staining on soft tissue sarcoma (STS) tissue microarrays. Relating to Fig. 1. STS tissue microarrays stained with anti-Endo180 mAb 39.10. Additional representative images of Endo180-positive and negative tumor cores for each STS subtype. Some tumor cores demonstrate Endo180-negative tumor cells interspersed with Endo180-positive cancer-associated fibroblasts (black arrowheads). Scale bars, 250 μm (liposarcomas), 100 μm (all other cores).

Supplementary Figure S1

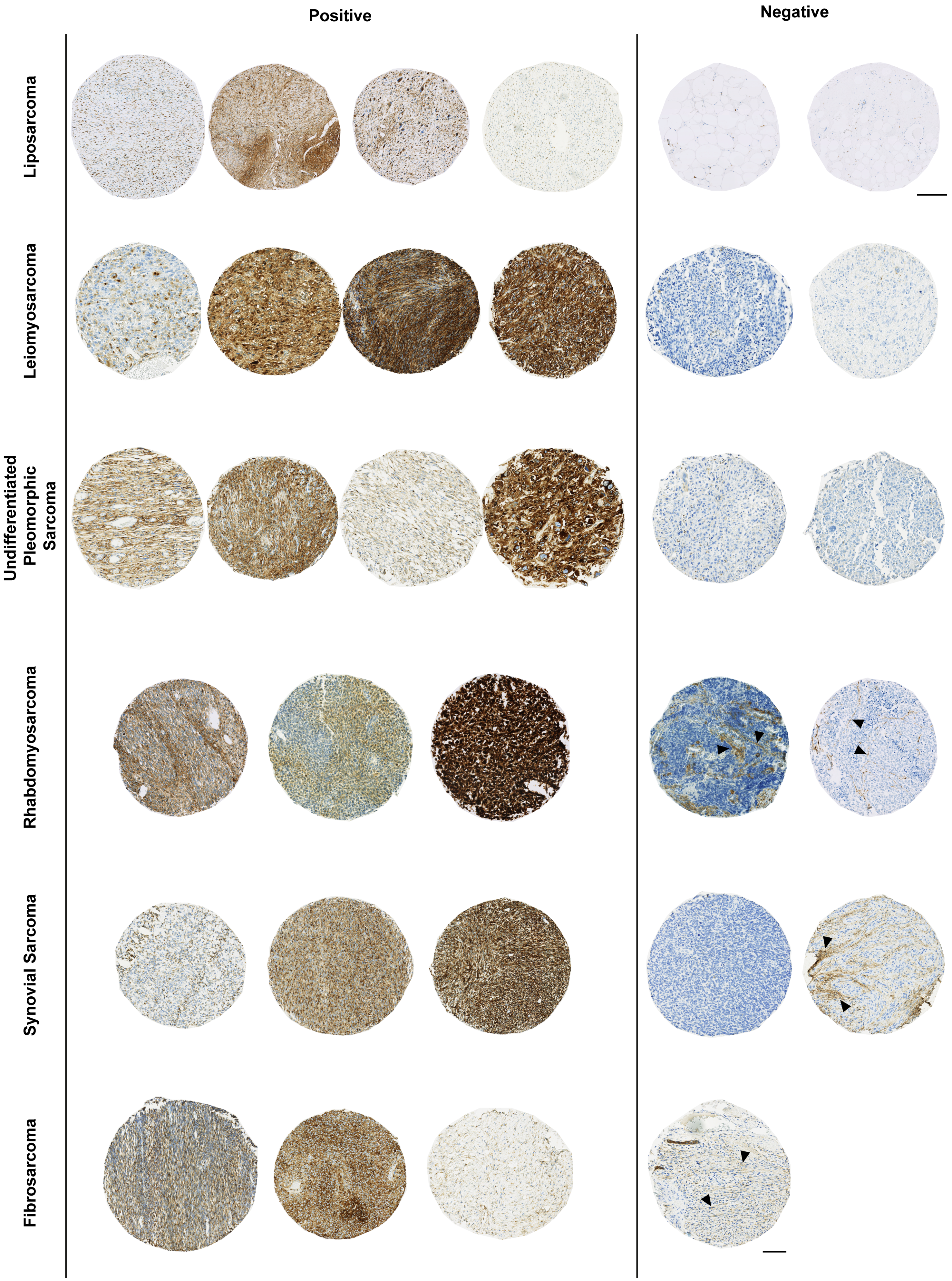

Supplement: Supplementary Figure S1 — Examples of Endo180 positive and negative staining in subtypes of soft tissue sarcoma tumor cores. [file mct-22-0312_supplementary_figure_s1_suppsf1.pdf]
