# Supplementary material for: Endo180 (MRC2) Antibody–Drug Conjugate for the Treatment of Sarcoma
Source: Mol Cancer Ther. 2022 Nov 18;22(2):240–53. doi: 10.1158/1535-7163.MCT-22-0312 (PMC9890142; doi:10.1158/1535-7163.MCT-22-0312)

Supplementary Figure S3. Endo180 expression and internalization in sarcoma cell lines. a. Representative confocal images of fixed and permeabilized sarcoma (MG-63, HT-1080, A-204, G-402, SK-UT-1) and epithelial cancer (MCF-7, HT-29) cell lines stained with A5/158 followed by Alexa488-conjugated anti-mouse IgG (green). Nuclei were counterstained with DAPI (blue). Scale bar, 50 µm. **b.** Relating to Fig. 3. Representative confocal images of sarcoma (MG-63 and HT-1080) and epithelial breast cancer (MCF-7) cell lines cultured with Isotype-488 (green) for 1 h at 4°C followed by incubation with LysoTracker Red (red) and Hoechst 33342 (blue) for 30 min at 37°C. Representative images out of two fields of view from experiments repeated on at least 3 occasions with equivalent findings. Scale bar, 50 µm.

Supplementary Fig. S3

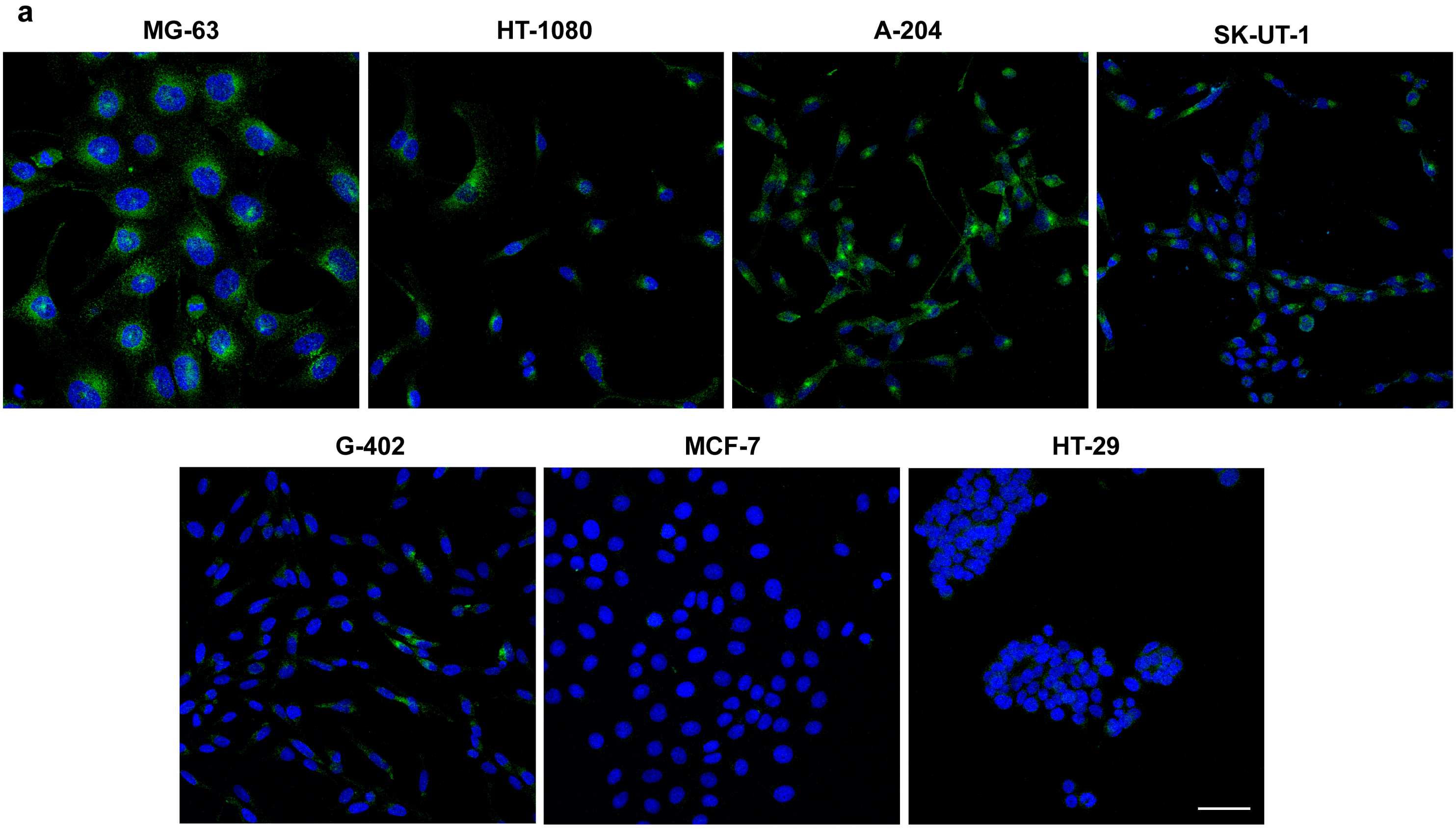

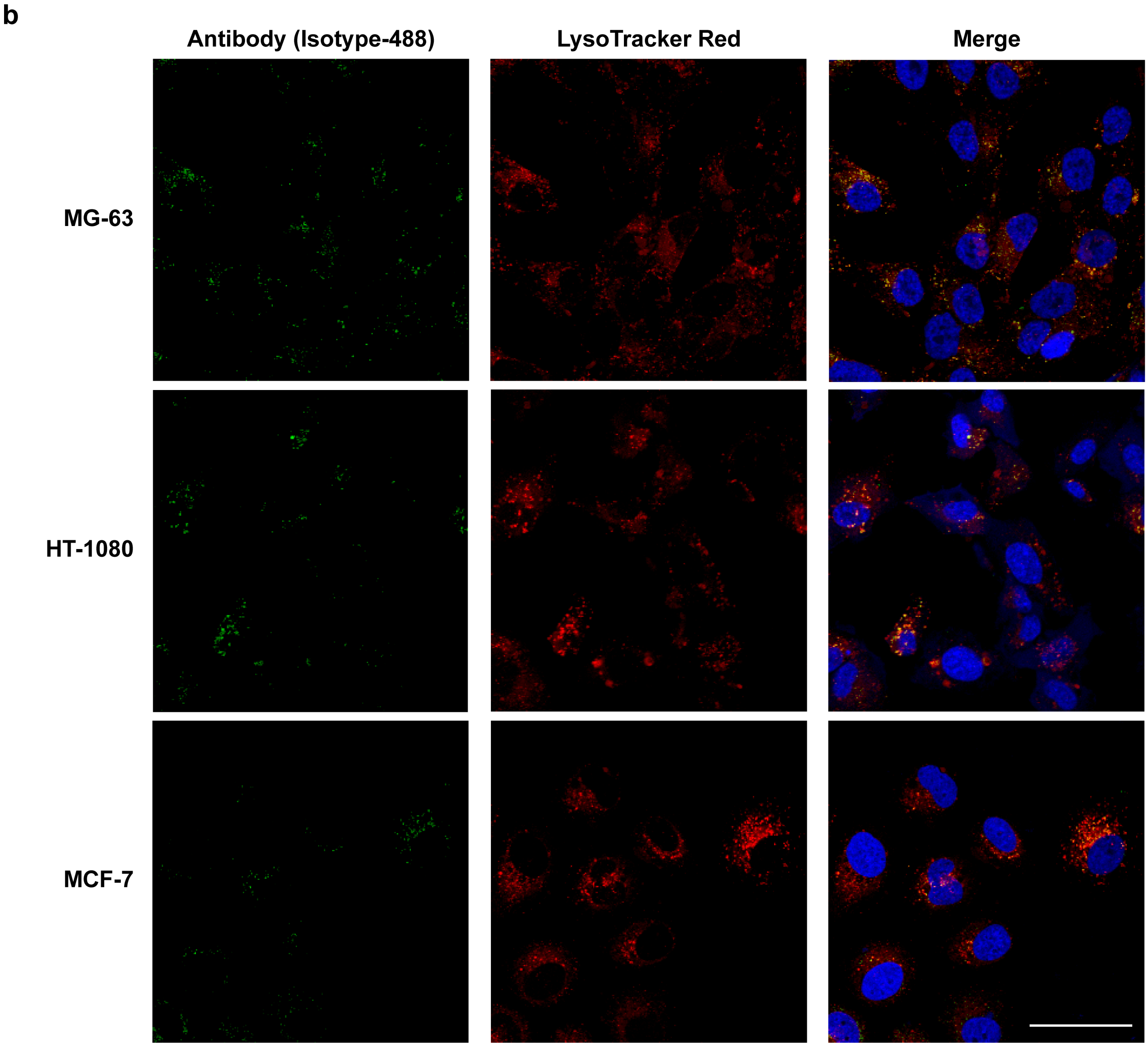

Supplement: Supplementary Figure S3 — Endo180 protein expression in sarcoma cell lines compared to epithelial cancer cells by immunofluorescence. Additionally, this figure shows the isotype control antibody conjugated to AlexaFluor 488 is not internalized into Endo180 expressing sarcoma cell lines and is not trafficked to the lysosome. [file mct-22-0312_supplementary_figure_s3_suppsf3.pdf]
