# Supplementary material for: Endo180 (MRC2) Antibody–Drug Conjugate for the Treatment of Sarcoma
Source: Mol Cancer Ther. 2022 Nov 18;22(2):240–53. doi: 10.1158/1535-7163.MCT-22-0312 (PMC9890142; doi:10.1158/1535-7163.MCT-22-0312)

Supplementary Figure S2. Endo180 expression in normal tissues and sarcoma datasets. Relating to Fig. 1. **a.** Representative images of normal human tissues stained with 39.10. Scale bars, 2.5 mm (whole tissue section), 100 µm (whole core), 50 µm (enlarged inset). **b.** *MRC2* expression in The Cancer Genome Atlas (TCGA) dedifferentiated liposarcoma (*n*=58), leiomyosarcoma (*n*=104), and undifferentiated pleiomorphic sarcoma (*n*=50) (violin plots, red lines indicate median expression levels, dotted lines indicate upper and lower quartiles, Kruskal-Wallis test, Dunn's multiple comparison test). **c.** *MRC2* expression in pediatric cancer (blue) and normal tissue (grey) from the NCI Oncogenomics data portal. Dotted line indicates mean *MRC2* expression across normal tissues. Data shown are mean values ±SEM.

Supplementary Fig. S2

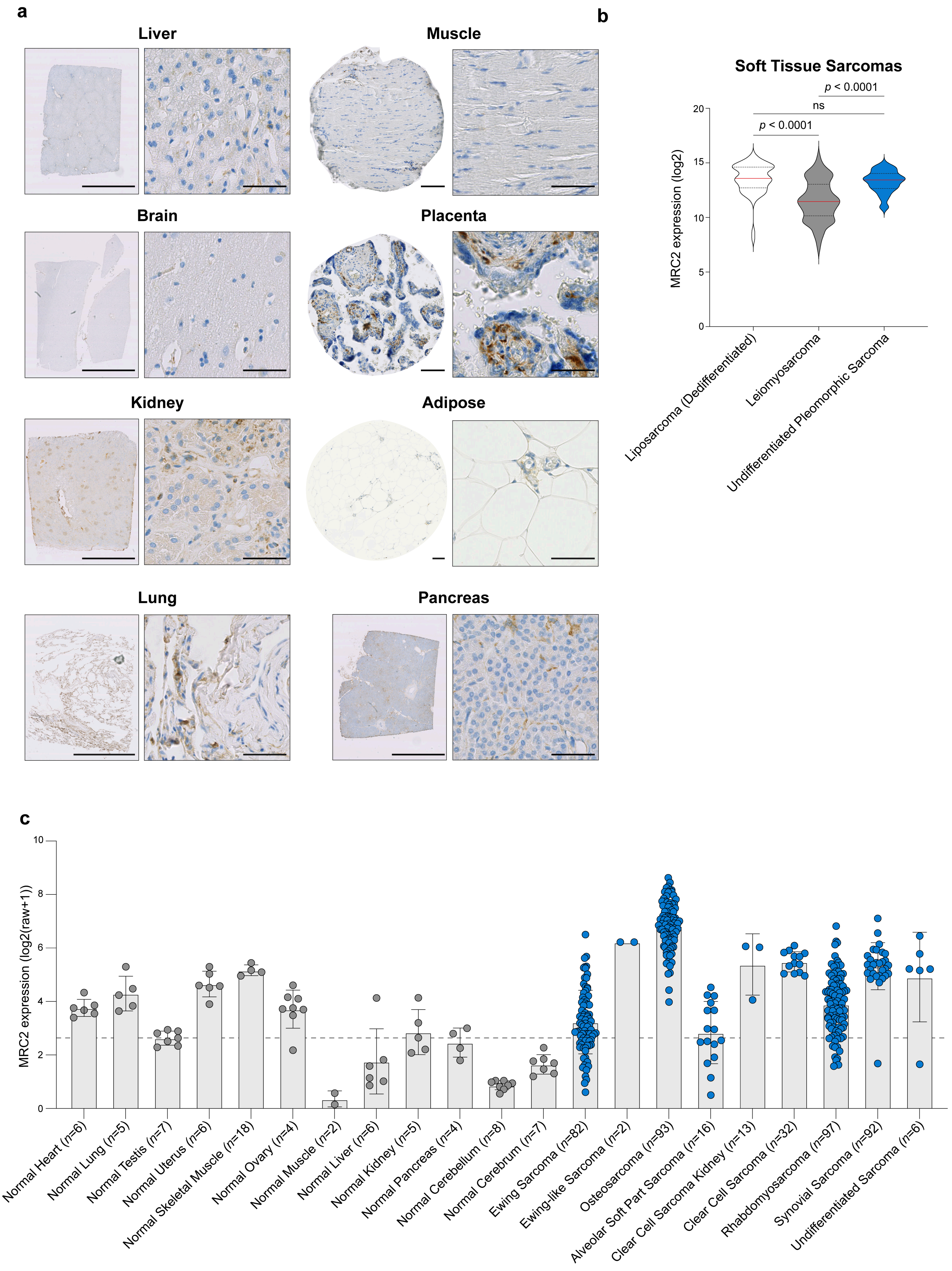

Supplement: Supplementary Figure S2 — Endo180 protein and gene expression in healthy human tissue compared to sarcoma subtypes. [file mct-22-0312_supplementary_figure_s2_suppsf2.pdf]
