# Supplementary material for: Endo180 (MRC2) Antibody–Drug Conjugate for the Treatment of Sarcoma
Source: Mol Cancer Ther. 2022 Nov 18;22(2):240–53. doi: 10.1158/1535-7163.MCT-22-0312 (PMC9890142; doi:10.1158/1535-7163.MCT-22-0312)

Supplementary Figure S4. Characterization of the vc-MMAE conjugated antibodies. Relating to Fig. 4. Anti-Endo180 mAb A5/158 and isotype control antibody were conjugated to MMAE using a cleavable valine-citrulline (vc) linker**. a.** Hydrophobic interaction chromatography was used to determine the final ADC composition**.** The resulting ADCs had a DAR of 4.0 and 3.4 for A5/158-vc-MMAE and Isotype-vc-MMAE, respectively. **b.** Size exclusion chromatography was used to assess aggregation of the conjugates. **c**. Western blot analysis of sarcoma (MG-63 and HT-1080) and epithelial (MCF-7 and HT-29) cell lines. Protein expression detected by A5/158 (top left panel) and A5/158-vc-MMAE (top right panel), isotype control antibody (bottom left panel) and Isotype-vc-MMAE (bottom right panel). Molecular size markers are in kDa.

Supplementary Fig. S4

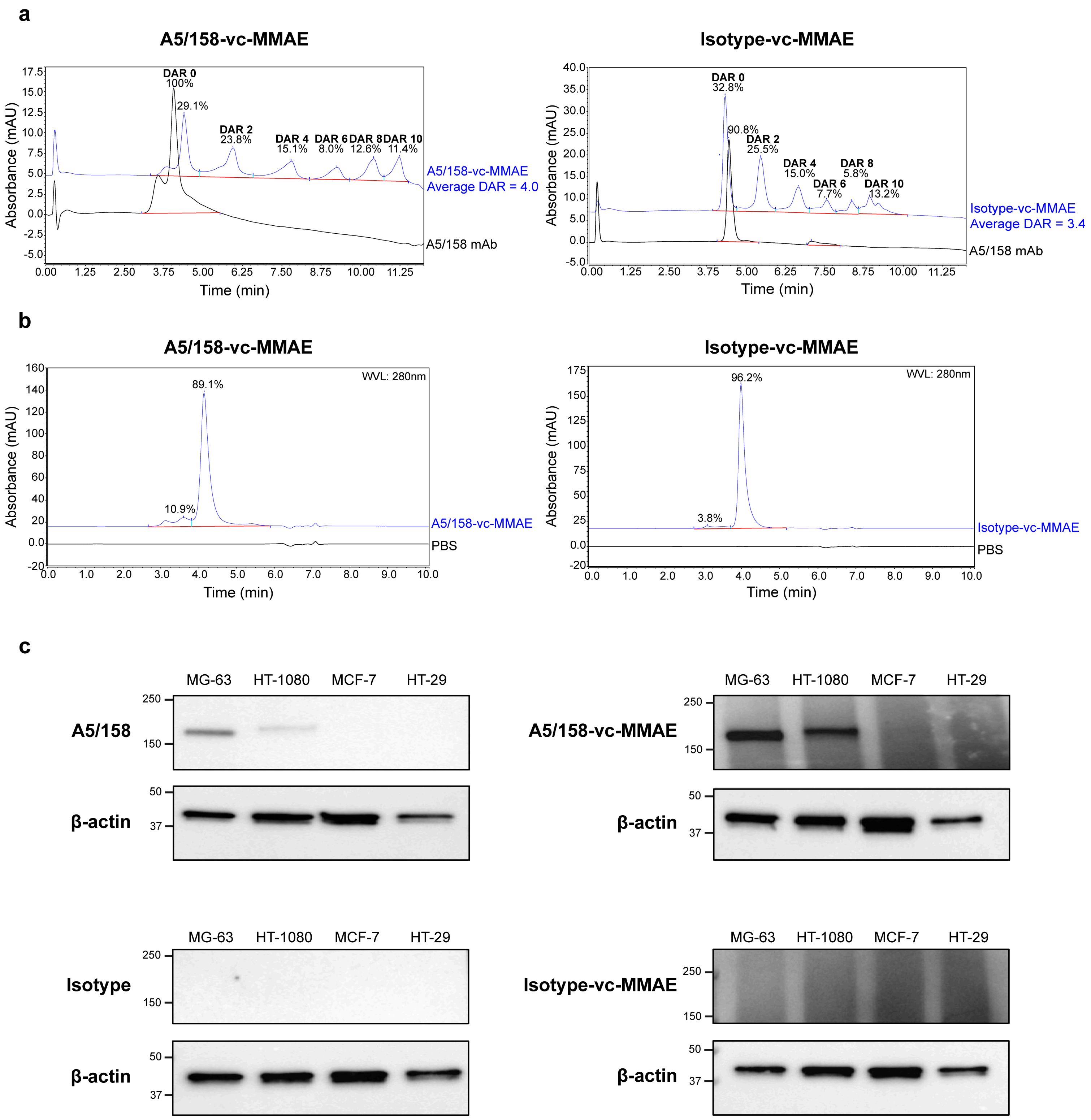

Supplement: Supplementary Figure S4 — Composition and drug to antibody ratio of A5/158 and the isotype control antibody conjugated to vc-MMAE. This figure additionally shows that that conjugation of A5/158 and the isotype control antibody to vc-MMAE does not induce antibody aggregation or disrupt Endo180 protein recognition. [file mct-22-0312_supplementary_figure_s4_suppsf4.pdf]
