# Supplementary material for: Endo180 (MRC2) Antibody–Drug Conjugate for the Treatment of Sarcoma
Source: Mol Cancer Ther. 2022 Nov 18;22(2):240–53. doi: 10.1158/1535-7163.MCT-22-0312 (PMC9890142; doi:10.1158/1535-7163.MCT-22-0312)

Supplementary Figure S5. Dose dependent *in vivo* activity of A5/158-vc-MMAE on tumor growth. Pilot *in vivo* experiment. **a.** Experimental schematic. 1 x 10^6 MG-63-mChLuc2 cells were injected subcutaneously (SC) into the flank of NSG mice. On day 10, once tumors reached ~3.5 mm in diameter, mice were randomized and began dose 1 of treatment. Table below shows treatment schedule for each mouse. Mice 1 and 2 were treated intravenously (IV) with vehicle (PBS) for 6 doses. Mice 2 and 3 received vehicle treatment until day 24, when average tumor diameter was 8.5 mm, and then treated with 5 mg/kg of A5/158-vc-MMAE on day 24 and day 27. Mice 5 and 6 were treated with 2.5 mg/kg or 5 mg/kg for 6 doses, respectively. Mouse 7 was treated with 10 mg/kg for 4 doses and then with vehicle on day 24 and day 27. All mice were culled on day 32 due to development of large lymph node metastases in the vehicle-alone treated group (Mice 1 and 2). Red arrows indicate treatment days. **b.** Bodyweight of individual mice relative to weight on day 0. **c.** Tumor growth of individual mice monitored by tumor diameter and volume. **d.** Growth rate of tumors**. e.** Final tumor volume and tumor weight. **f**. Immunohistochemistry images of tumors stained with 39.10. Scale bar, 2.5 mm.

Supplementary Fig. S5

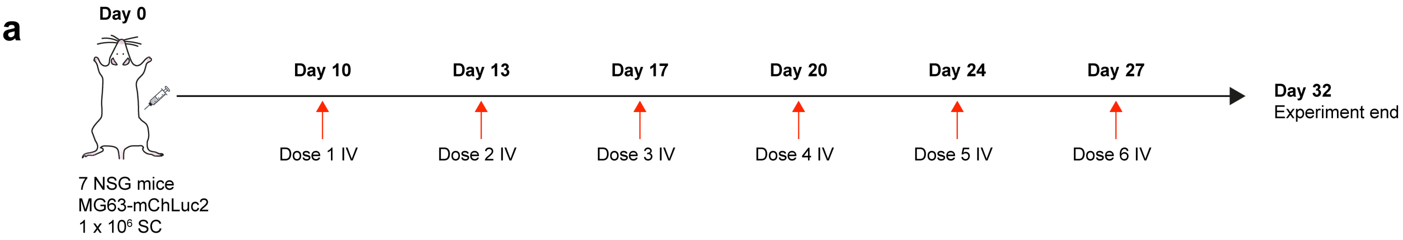

	Day 10	Day 13	Day 17	Day 20	Day 24	Day 27
**Mouse**	**Dose 1 (mg/kg)**	**Dose 2 (mg/kg)**	**Dose 3 (mg/kg)**	**Dose 4 (mg/kg)**	**Dose 5 (mg/kg)**	**Dose 6 (mg/kg)**
1	Vehicle	Vehicle	Vehicle	Vehicle	Vehicle	Vehicle
2	Vehicle	Vehicle	Vehicle	Vehicle	Vehicle	Vehicle
3	Vehicle	Vehicle	Vehicle	Vehicle	5	5
4	Vehicle	Vehicle	Vehicle	Vehicle	5	5
5	2.5	2.5	2.5	2.5	2.5	2.5
6	5	5	5	5	5	5
7	10	10	10	10	Vehicle	Vehicle

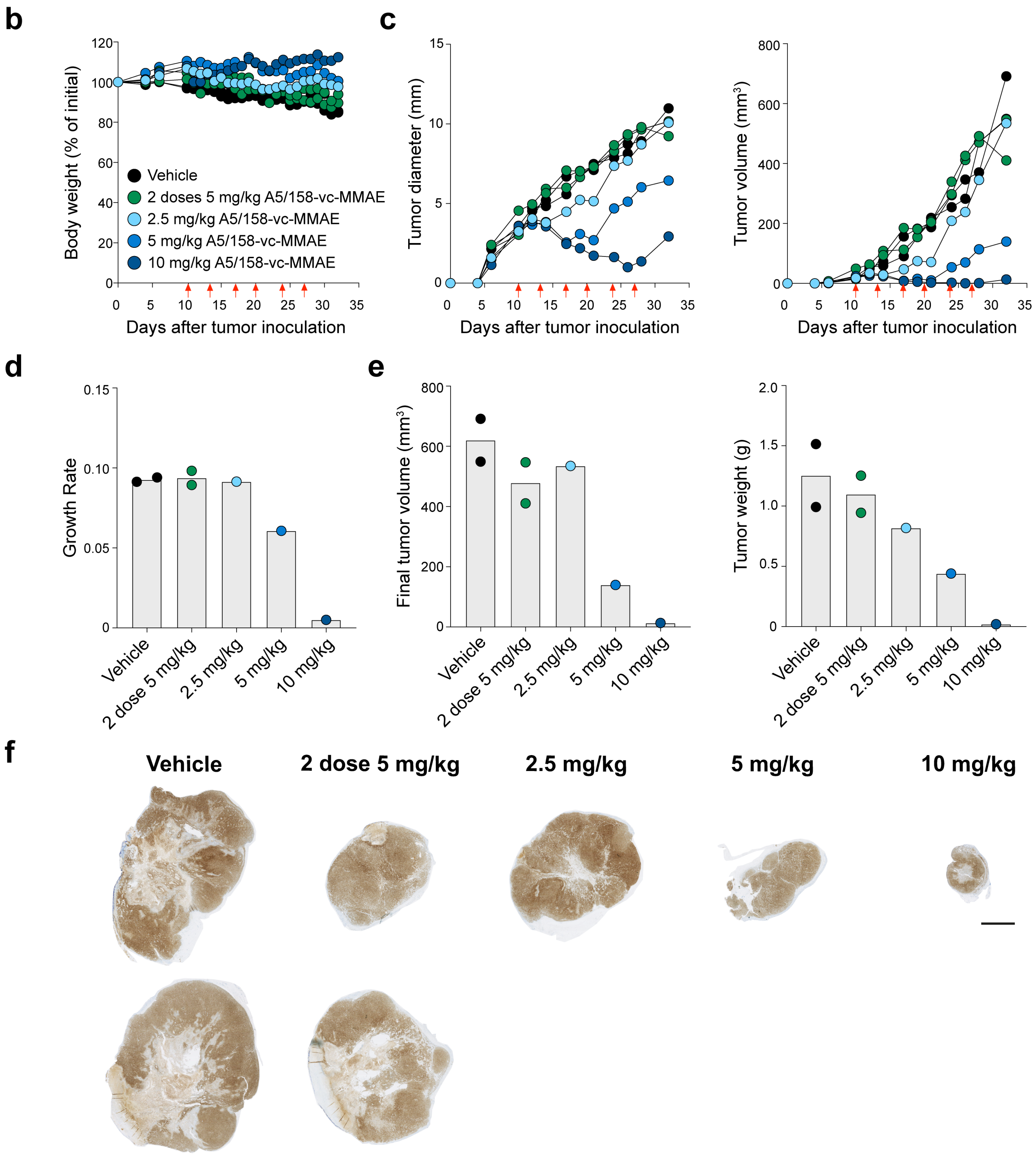

Supplement: Supplementary Figure S5 — In vivo pilot experiment of NSG mice bearing MG-63 subcutaneous tumours treated intravenously with different concentrations of A5/158-vc-MMAE. [file mct-22-0312_supplementary_figure_s5_suppsf5.pdf]
