# Supplementary material for: Endo180 (MRC2) Antibody–Drug Conjugate for the Treatment of Sarcoma
Source: Mol Cancer Ther. 2022 Nov 18;22(2):240–53. doi: 10.1158/1535-7163.MCT-22-0312 (PMC9890142; doi:10.1158/1535-7163.MCT-22-0312)

Supplementary Figure S6. Dose dependent *in vivo* activity of A5/158-vc-MMAE on metastasis. Continuation of Supplementary Fig. S5. Lung and liver FFPE sections were stained for human lamin A/C. **a.** Stained lung sections. Scale bar, 2.5 mm. **b**. Quantification of the number of lung macrometastatic lesions, average area of individual lung lesions and % metastatic lung tumor area. **c.** Stained liver sections. Scale bar, 5 mm.

Supplementary Fig. S6

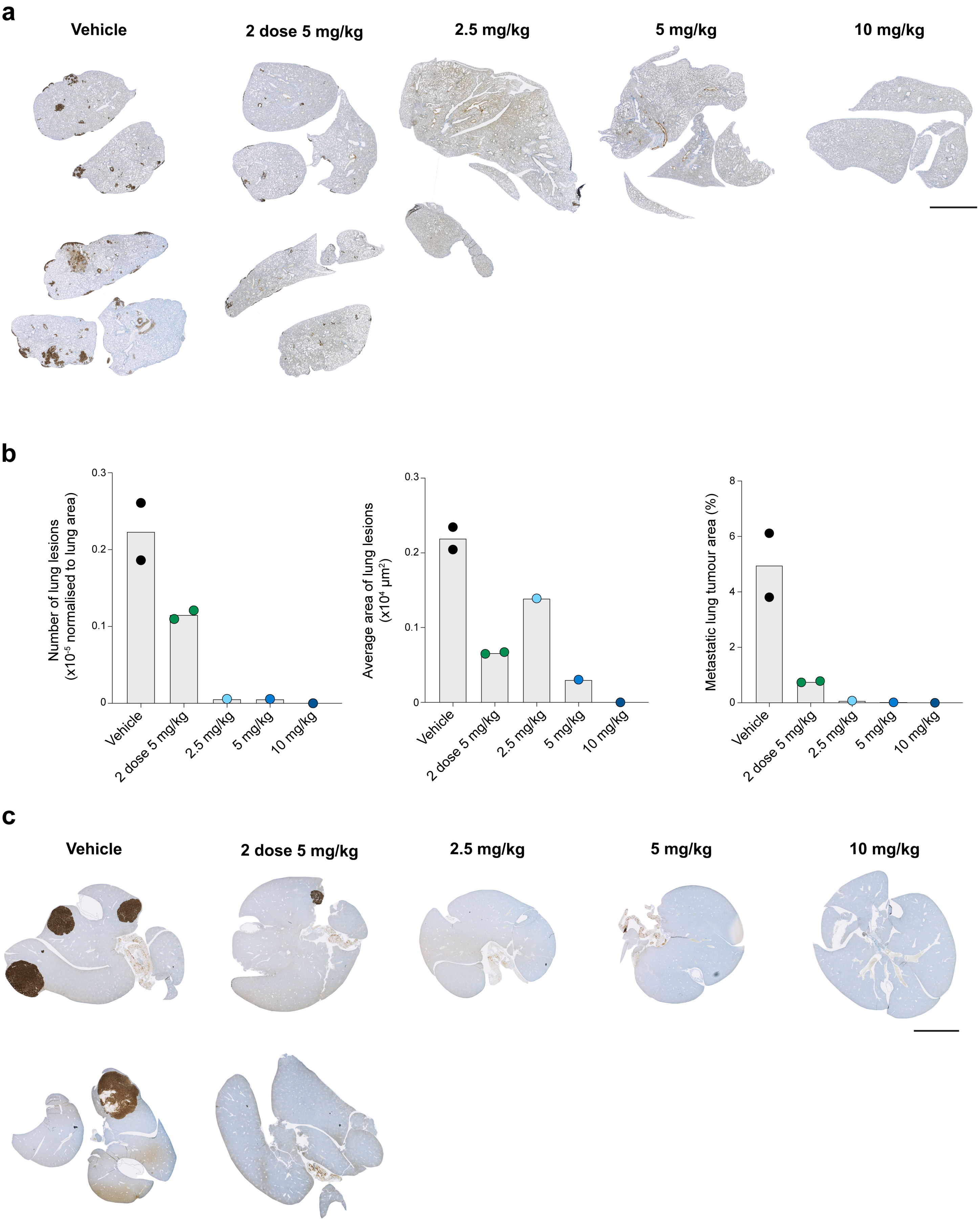

Supplement: Supplementary Figure S6 — Continuation of Figure S5 showing the effect of A5/158-vc-MMAE treatment on the spontaneous metastatic spread of MG-63 cells to the lungs and liver. [file mct-22-0312_supplementary_figure_s6_suppsf6.pdf]
