# Supplementary material for: Endo180 (MRC2) Antibody–Drug Conjugate for the Treatment of Sarcoma
Source: Mol Cancer Ther. 2022 Nov 18;22(2):240–53. doi: 10.1158/1535-7163.MCT-22-0312 (PMC9890142; doi:10.1158/1535-7163.MCT-22-0312)

Supplementary Figure S7. A5/158-vc-MMAE decreases the number and size of spontaneous liver metastases. FFPE liver sections from Fig. 5 and 6 were stained for human lamin A/C. **a.** Immunohistochemistry images. Scale bar, 5 mm. **b.** Quantification of the number of macrometastatic liver lesions, average area of liver lesions and % metastatic liver tumor area (mean values of 3 sections per liver). Data shown are mean values per mouse ±SEM (two-sided Mann-Whitney *U* test).

Supplementary Fig. S7

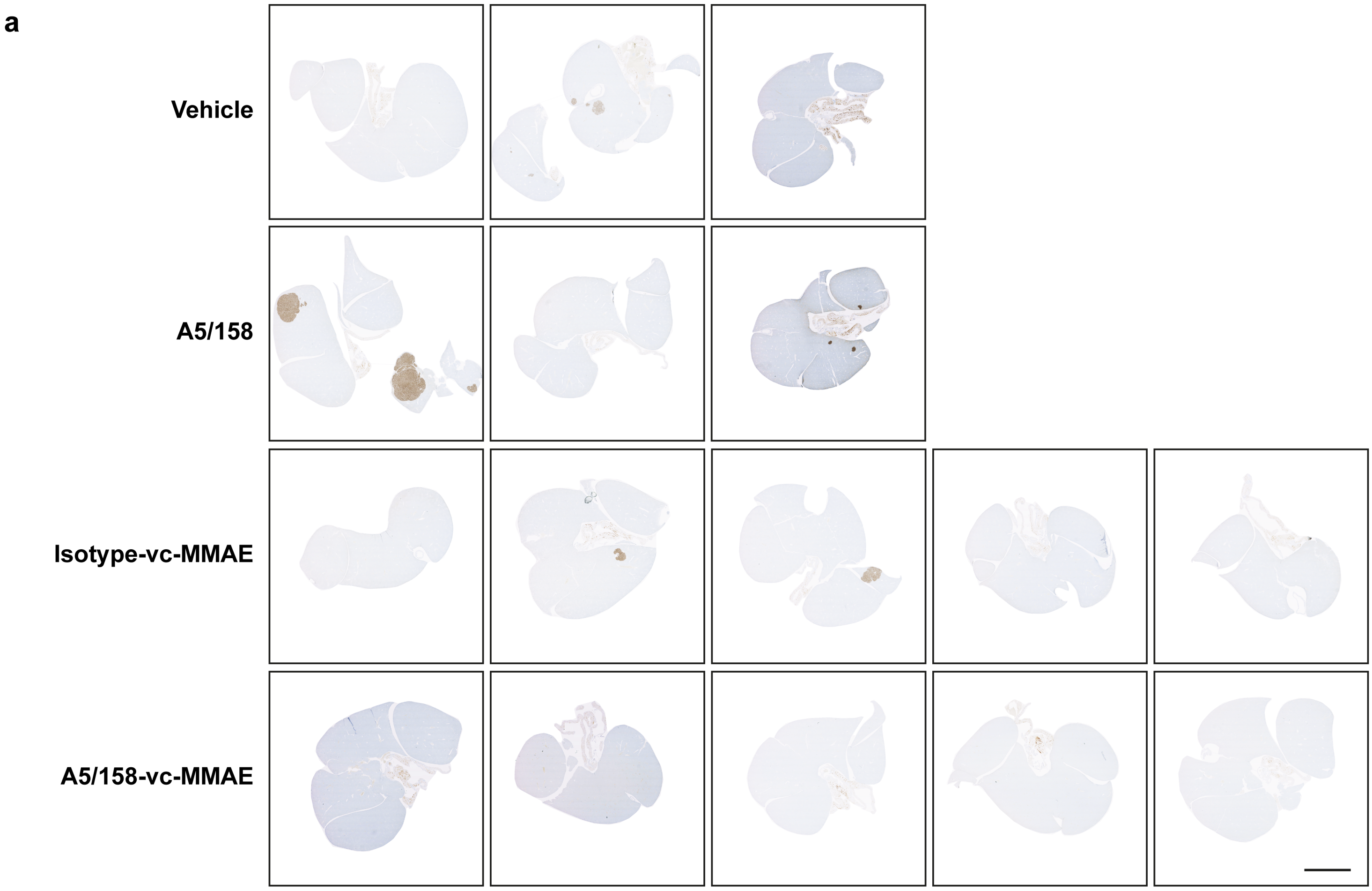

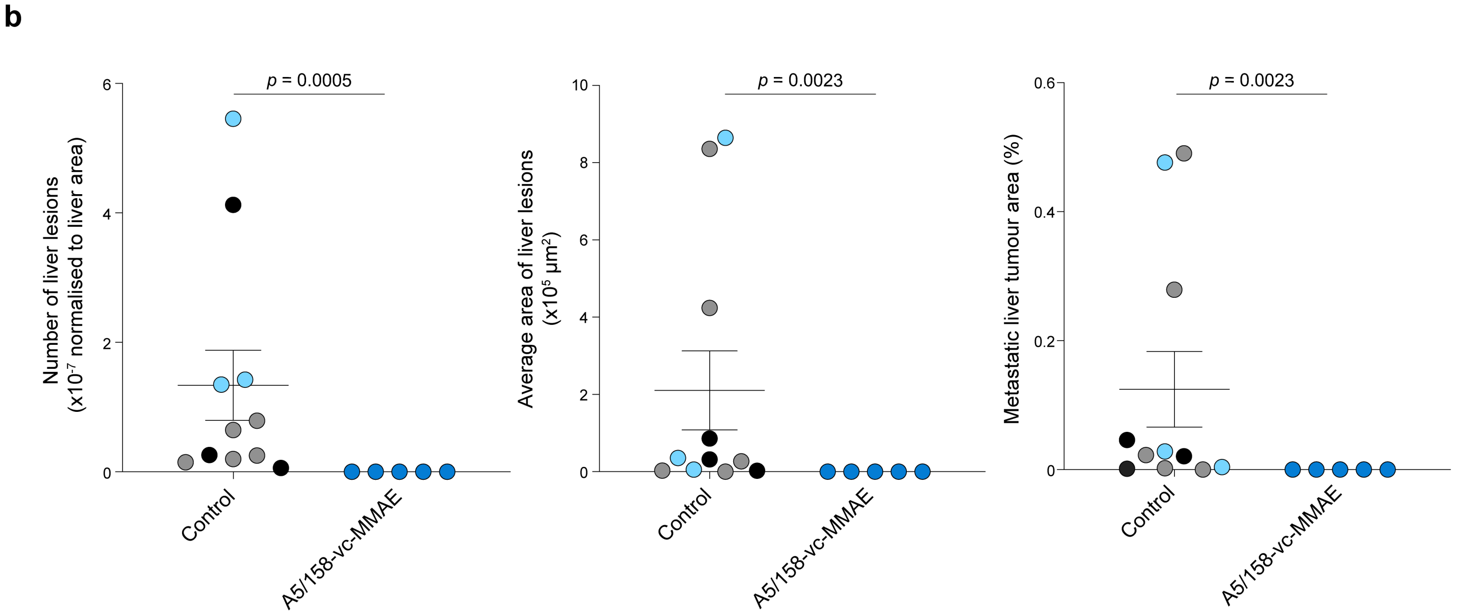

Supplement: Supplementary Figure S7 — Immunohistochemical staining and quantification of the effect of A5/158, A5/158-vc-MMAE and Isotype-vc-MMAE treatment on the spontaneous metastasis of MG-63 cells to the liver in NSG mice. [file mct-22-0312_supplementary_figure_s7_suppsf7.pdf]
